# Vitamin A Oral Supplementation Induces Oxidative Stress and Suppresses IL-10 and HSP70 in Skeletal Muscle of Trained Rats

**DOI:** 10.3390/nu9040353

**Published:** 2017-04-02

**Authors:** Lyvia Lintzmaier Petiz, Carolina Saibro Girardi, Rafael Calixto Bortolin, Alice Kunzler, Juciano Gasparotto, Thallita Kelly Rabelo, Cristiane Matté, José Claudio Fonseca Moreira, Daniel Pens Gelain

**Affiliations:** Departamento de Bioquímica, Instituto de Ciências Básicas da Saúde, Universidade Federal do Rio Grande do Sul, 90035-000, Porto Alegre, Brazil; carolsg94@hotmail.com (C.S.G.); rafaelbortolin@hotmail.com (R.C.B.); alice.bio@hotmail.com (A.K.); juciano.gasparotto@gmail.com (J.G.); talitabioq@gmail.com (T.K.R.); cristianematte@gmail.com (C.M.); jcfm@ufrgs.br (J.C.F.M.); dgelain@yahoo.com.br (D.P.G.)

**Keywords:** antioxidant enzymes, antioxidant supplements, exercise, cytokines, vitamin

## Abstract

Exercise training intensity is the major variant that influences the relationship between exercise, redox balance, and immune response. Supplement intake is a common practice for oxidative stress prevention; the effects of vitamin A (VA) on exercise training are not yet described, even though this molecule exhibits antioxidant properties. We investigated the role of VA supplementation on redox and immune responses of adult Wistar rats subjected to swimming training. Animals were divided into four groups: sedentary, sedentary + VA, exercise training, and exercise training + VA. Over eight weeks, animals were submitted to intense swimming 5 times/week and a VA daily intake of 450 retinol equivalents/day. VA impaired the total serum antioxidant capacity acquired by exercise, with no change in interleukin-1β and tumor necrosis factor-α levels. In skeletal muscle, VA caused lipid peroxidation and protein damage without differences in antioxidant enzyme activities; however, Western blot analysis showed that expression of superoxide dismutase-1 was downregulated, and upregulation of superoxide dismutase-2 induced by exercise was blunted by VA. Furthermore, VA supplementation decreased anti-inflammatory interleukin-10 and heat shock protein 70 expression, important factors for positive exercise adaptations and tissue damage prevention. Our data showed that VA supplementation did not confer any antioxidative and/or protective effects, attenuating exercise-acquired benefits in the skeletal muscle.

## 1. Introduction

Benefits generated by regular physical exercise on human health are well known. Regular physical activity is recommended for the prevention and treatment of metabolic syndrome diseases, such as high blood pressure and type 2 diabetes [1]. Moderate to intense physical activity exerts a large influence on redox balance and immunity modulation [2,3]. Due to high oxygen demand by skeletal muscle, exercise increases the generation of reactive oxygen species (ROS), such as the superoxide anion radical, hydrogen peroxide, and the hydroxyl radical [4,5]. At physiological concentrations, ROS act as signaling molecules that lead to positive adaptations induced by exercise, such as upregulation of endogenous antioxidant defenses, skeletal muscle hypertrophy, and mitochondrial biogenesis [6,7,8]. When the redox imbalance intensifies towards an excessive pro-oxidant state, ROS may cause DNA damage, functional loss of protein structures, such as enzymes and membrane receptors, and structural damage of the cell lipid bilayer [9]. In athletes, chronic oxidative stress can lead to performance decline, fatigue, muscular damage, and overtraining [10]. Furthermore, levels of inflammatory cytokines rise considerably after vigorous exercise training, which is often related to ROS overload [11]. This can lead to immune improvement or depression, and the outcome is determined by training intensity. The relationship between exercise and susceptibility to illness is described by a “J curve” concept. It suggests that, while individuals that regularly perform moderate intensity exercise improve their immune system, excessive bouts of prolonged training can impair immune function [12]. This results in responses such as release of pro-inflammatory cytokines, such as tumor necrosis factor α (TNF-α) and interleukin-1β (IL-1β), and anti-inflammatory cytokines, such as interleukin-6 (IL-6) and interleukin-10 (IL-10) [13]. 

Besides the endogenous defenses, other factors like nutrition can exert a major role in the prevention of oxidative stress and immunity depression [14]. Polyphenols found in fruits, plants, and vegetables demonstrate antioxidant potential in the circulatory system, although the molecular basis of how they affect exercise training remains unclear [5]. Supplementation with vitamins is also widely used by athletes to avoid skeletal muscle injury, especially vitamin C and E [15,16]; however, these benefits remain questionable in the literature. Studies show different outcomes from the combination of vitamins C and E, such as no effect during exercise training [15,17], reduced lipid and protein damage after eccentric exercise [18,19], and decreased stress markers without antioxidant benefits [20].

Another vitamin that has shown to be involved in the redox process is vitamin A (VA), a fat-soluble vitamin obtained from different compounds: all-*trans* retinol (considered the VA molecule), β-carotene (VA precursor), and retinyl esters (retinol esterified to other molecules, such as palmitate) [21]. It is essential to the correct functions of several metabolic and physiological processes, such as vision, hematopoiesis, embryogenic development, cell differentiation, gene transcription, and the immune system [22]. The arrangement of long chains of conjugated double bonds, common to all retinoids, allows the structure to exert ROS scavenging properties, and usually, this activity is involved in the prevention of lipid peroxidation [23,24,25]. However, retinol has been observed to present moderate to low antioxidant activity, and VA supplementation has been associated with some adverse effects. Our group has previously shown that oral retinyl palmitate supplementation induces, in fact, a pro-oxidant environment in several tissues, including the heart, brain, and lungs of Wistar rats [26,27,28]. Moreover, it was previously described that mice fed with retinyl palmitate in low doses developed aortic valve stenosis and calcification [29]. A clinical study of the effect of a combined supplement of β-carotene and retinyl palmitate on lung cancer prevention actually revealed harmful effects, as it increased the incidence of lung cancer and cardiovascular diseases in smokers and workers exposed to asbestos [30]. Reviews that address the effects of supplementation on exercise-induced oxidative stress often mention VA or its precursor β-carotene as a potential antioxidant molecule [4,31]. However, its effects on exercise training are poorly documented, and mechanisms in vivo remain unclear. Here, we evaluated the effect of VA supplementation, given in the form of retinyl palmitate, on parameters of oxidative stress and inflammation in rats subjected to exercise training, to determine if VA enhances the benefits conferred by regular exercise. The dose of choice for VA treatment was 450 retinol equivalents (RE)/day. We calculated the human equivalent doses (HED) using the dose-by-factor approach [32], with values based on the daily recommendation for adults. We considered the daily recommendation of 800 RE [33] and the fact that VA is provided in the diet as it is present in the standard chow in amounts meeting the daily requirement for this vitamin. We chose this approach to avoid hypervitaminosis or other effects caused by excessive VA intake, since higher doses of VA produce deleterious effects on the brain, lungs, and cardiovascular systems as mentioned above. This is the first study describing the effects of chronic aerobic exercise training and VA supplementation on redox and immunity parameters on skeletal muscle.

## 2. Materials and Methods

The Ethical Committee for Animal Experimentation of the Federal University of Rio Grande do Sul (CEUA-UFRGS) granted the approval for this project under the number 25837, and all experiments were conducted under the National Institute of Health Guide for the Care and Use of Laboratory Animals (2011) [34]. Protocols also followed the guidelines of the Brazilian Society of Animal Science Experimentation (SBCAL). This study complied with the 3Rs principle: replacement of animals by alternatives wherever possible; reduction in the number of animals used; and refinement of experimental conditions and procedures to minimize the harm to the animals.

### 2.1. Animals

Thirty-two adult male Wistar rats (7 weeks old, weight 250–300 g) were provided by our breeding colony. During one week, animals were manipulated for adaptation. Animals were maintained in cages in a room with an ambient temperature of 22 ± 1 °C and a 12/12 h light/dark cycle, with access to food and water ad libitum.

### 2.2. Swimming Exercise Training Protocol

**Figure 1:**
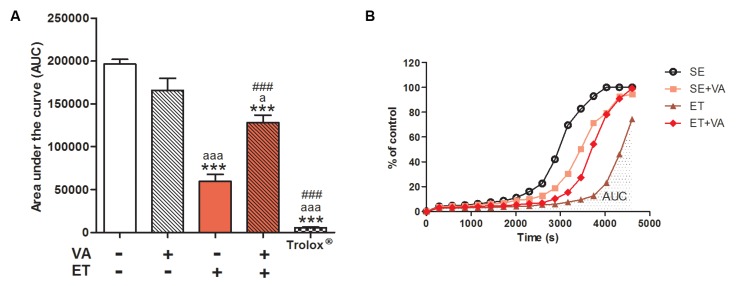
(**A**) Total reactive antioxidant potential (TRAP) from serum. Data presented as mean ± SEM (*n* = 6–8); (**B**) demonstrative reaction kinetics of TRAP. Control: peroxyl radical system that generates luminescence at a steady rate (considered 100% of free radical production). Luminescence generated by this system in the presence of samples is monitored through time. Trolox = antioxidant applied as positive control (100 µM). VA: vitamin A; ET: exercise training. *** *p* < 0.001 significant difference from sedentary group; ^a^ *p* < 0.05; ^aaa^ *p* < 0.001 significant difference from sedentary + vitamin A group; ^###^ *p* < 0.001 significant difference from exercise training group using one-way ANOVA followed by Tukey’s post hoc test.

The training protocol lasted 8 weeks in total (Figure 1). For the first week, animals remained in shallow water for 20–60 min each day. Next, animals were randomized into four groups: sedentary (SE), sedentary supplemented with vitamin A (SE + VA), exercise training (ET), and exercise training supplemented with vitamin A (ET + VA). In the following two weeks, the swimming protocol started with 10 min/day, gradually increasing to 60 min/day. The exercise protocol was conducted between 6 and 8 pm, in a specific swimming tank for rodents with water at 31 ± 1 °C. Over the following 5 weeks, training consisted of 60 min/day, 5 days/week [35]. Once a week, animal weight values were utilized to calculate the overload (0, 2, 4, 6% body weight). To minimize water-induced stress differences between groups, sedentary animals were placed in shallow water for 20 min 5 days/week during the 8 weeks. After each session, animals were towel-dried and returned to their cages.

### 2.3. Vitamin A Supplementation

Throughout the protocol period (8 weeks), animals from groups SE + VA and ET + VA had a daily intake of 450 RE (1500 IU)/kg/day of retinyl palmitate (Arovit, Bayer, Rio de Janeiro, RJ, Brazil). We calculated the human equivalent doses (HED) using the dose-by-factor approach [32]. Arovit presents a water-soluble form of vitamin A, allowing use of saline as a vehicle solution. Supplementation was orally administered via gavage, in a maximum volume of 0.5 mL. Groups SE and ET received only the vehicle.

### 2.4. Tissue Preparation

Twenty-four hours after the last session of exercise and vitamin A supplementation animals were euthanized by decapitation. Blood samples were immediately centrifuged for serum separation. Vastus medialis skeletal muscle was removed and stored at −80 °C. For biochemical analysis, tissue was homogenized in phosphate buffer (PB) and centrifuged (3000× *g*, 10 min), and sample supernatant was used for analysis. Protein content was quantified by the Lowry method [36] using bovine serum albumin (BSA) as a standard. For Western blotting, tissue was homogenized in RIPA buffer (20 mM Tris-HCl pH 7.5; 150 mM NaCl; 1 mM ethylenediamine tetra-acetic acid (EDTA); 1 mM ethylene glycol tetra-acetic acid (EGTA); 2.5 mM sodium pyrophosphate; 1% sodium deoxycholate; 1% Tergitol-type NP-40; 1 mM β-glycerophosphate; 1 mM sodium orthovanadate; 1 µg/mL leupeptin), centrifuged, and the homogenate was added to Laemmli-buffer (62.5 mM Tris-HCl pH 6.8; 1% SDS; 10% glycerol) with 5% β-mercaptoethanol.

### 2.5. Serum Analysis

Serum activity of enzymes creatine kinase (CK) and lactate dehydrogenase (LDH) was measured with commercial kits (Labtest, São Paulo, Brazil). Total reactive antioxidant potential (TRAP) was determined as described in the literature [37]. The assay is based on the employment of a peroxyl radical generator (2,2-azo-bis(2-amidinopropane); AAPH) mixed with luminol, and the scavenging activity of samples prevents luminol oxidation by AAPH. The synthetic antioxidant Trolox (Acros Organics BVBA, Geel, Belgium), a vitamin E analog, was applied as a positive control at a concentration of 100 µM [38]. The antioxidant capacity of samples was recorded through 60 min and results were calculated as area under the curve (AUC). Quantitative analysis of IL-1β and IL-10 was determined by indirect ELISA using polyclonal antibodies (Abcam, Cambridge, UK). TNF-α was quantified using an ELISA sandwich kit following the manufacturer’s instructions (R&D Systems, Inc., Minneapolis, MN, USA).

### 2.6. Skeletal Muscle Analysis

#### 2.6.1. Oxidative Stress Parameters

Lipid peroxidation was detected through measuring thiobarbituric acid reactive species (TBARS) levels [39]. Samples deproteinized by 10% trichloroacetic acid (TCA) were heated at 100 °C for 25 min with 0.67% thiobarbituric acid, and TBARS were quantified spectrophotometrically at a wavelength of 532 nm. Protein damage was quantified by carbonyl group detection [40]. The technique involves incubating sample proteins, previously precipitated with 20% TCA, with 2,4-dinitrophenylhydrazine (DNPH), and quantification at a wavelength of 370 nm. Thiol content was quantified in protein-containing and non-protein-containing (after acid-induced precipitation) samples through an Ellman’s assay [41]. Samples were diluted in phosphate-buffered saline (PBS) and incubated with 10 mM of 5,5-dithiobis(2-nitrobenzoic) (DTNB) for 60 min at room temperature of 23 ± 1 °C. Quantification was performed using a spectrophotometer at a wavelength of 412 nm.

#### 2.6.2. Activity of Antioxidant Enzymes

Determination of the activities of antioxidant enzymes superoxide dismutase (SOD), catalase (CAT), and glutathione peroxidase (GPx) was performed using spectrophotometric kinetics. CAT (EC 1.11.1.6) activity was measured by the decrease of hydrogen peroxide (H_2_O_2_) followed by measurement at a UV wavelength of 240 nm [42]. SOD (EC 1.15.1.1) activity was measured indirectly by inhibition of adrenaline auto-oxidation, measured at 480 nm [43]. GPx (EC 1.11.1.9) activity was evaluated by the decrease of nicotinamide adenine dinucleotide phosphate reduced form (NADPH) in the presence of glutathione (GSH), *tert*-butyl hydroperoxide, and glutathione reductase, measured at 340 nm [44].

#### 2.6.3. Western Blotting

Samples from skeletal muscle (25 µg), run on an SDS-PAGE gel, were transferred to a nitrocellulose membrane (Millipore, Bedford, MA, USA) through semi-dry transference, and protein content confirmed using Ponceau S staining. After three cycles of TTBS (Tris 100 mM pH 7.5; 0.9% NaCl, and 0.1% Tween-20) washing, membranes were blocked with 5% non-fat dry milk for 1 h at room temperature. After washing, membranes were incubated with primary antibodies for SOD1, SOD2, CAT, IL-1β, IL-10, TNF-α, heat-shock protein 70 (HSP70), and β-actin for 2 h at room temperature in a 1:1000 dilution range. Secondary antibodies (anti-rabbit/mouse/goat peroxidase-linked—Cell Signaling Technology, Beverly, MA, USA) were incubated for 1 h at room temperature in a 1:2000 dilution range. Detection of immunoreactivity was performed through chemiluminescence using a Supersignal West Pico Chemiluminescent kit (Thermo Scientific, Rockford, IL, USA). Densitometry analysis was conducted with ImageJ software (version 1.50i, National Institutes of Health, Bethesda, MD, USA), and the results were expressed as ratio of protein:β-actin.

### 2.7. Statistics

All analyses and graphics were performed using GraphPad Prism (version 5.0, GraphPad Software Inc., San Diego, CA, USA). For comparison of four groups, one-way ANOVA followed by Tukey’s post hoc test was applied, and data expressed as the mean ± standard error (SEM) or median and interquartile. Differences were considered significant when *p* < 0.05.

## 3. Results

### 3.1. Protocol and Supplementation Effect on Total Body Weight

The ET group exhibited a significant reduction in body weight gain when compared to both sedentary groups (SE and SE + VA), probably due to the intense exercise protocol (Table 1). ET + VA group weight gain did not differ from the ET group, demonstrating that VA supplementation did not affect this parameter.

### 3.2. Serum Results

#### 3.2.1. Tissue Damage Markers

The cytosolic enzymes LDH and CK are expressed in myocytes, and detection of unusual activity in serum indicates tissue injury, especially skeletal muscle damage [45]. Table 2 displays the serum activity of these markers. LDH activity significantly increased in ET and ET + VA samples compared to SE, although no differences were detected between both exercised groups. CK activity did not change in exercised groups compared to SE, although the SE + VA group showed lower CK activity compared to SE.

#### 3.2.2. Redox Balance

The serum total antioxidant profile was assessed by the TRAP assay (Figure 1). The SE + VA group did not display a significant difference in serum antioxidant potential compared to SE. The ET group presented a high antioxidant profile, as expected since regular exercise improves endogenous antioxidant capacity [8]. However, the ET + VA group showed a significant reduction in antioxidant potential (approximately 50%), suggesting that vitamin A supplementation attenuates the antioxidant effect of exercise training on serum.

#### 3.2.3. Inflammation Markers

**Figure 2:**
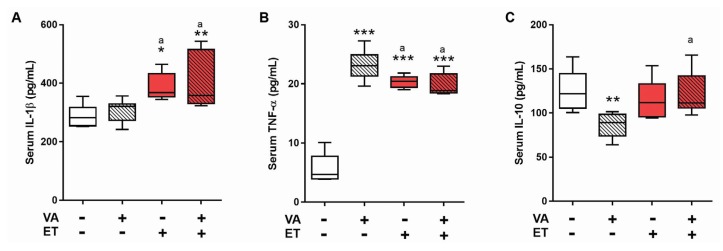
Levels of cytokines detected in serum by ELISA. Data presented as box (median) and whiskers (interquartile interval) diagram (*n* = 6–8). (**A**) Interleukin-1β; (**B**) Tumor necrosis factor-α; and (**C**) Interleukin-10. VA: vitamin A; ET: exercise training. * *p* < 0.05; ** *p* < 0.01; *** *p* < 0.001 significant difference from sedentary group; ^a^ *p* < 0.05 significant difference from sedentary + vitamin A group using one-way ANOVA followed by Tukey’s post hoc test.

Levels of pro-inflammatory cytokines IL-1β and TNF-α were significantly higher in ET and ET + VA groups when compared to SE, but these groups displayed no differences between each other, indicating that VA supplementation has no effect on modulation of IL-1β and TNF-α by ET (Figure 2A,B). Interestingly, VA supplementation in sedentary animals (SE + VA) induced a significant increase in serum TNF-α (Figure 2B). Levels of the anti-inflammatory cytokine IL-10 did not increase in any group, and the SE + VA group exhibited a significant decrease in IL-10 compared to SE (Figure 2C).

### 3.3. Skeletal Muscle

#### 3.3.1. Oxidative Stress Markers

**Figure 3:**
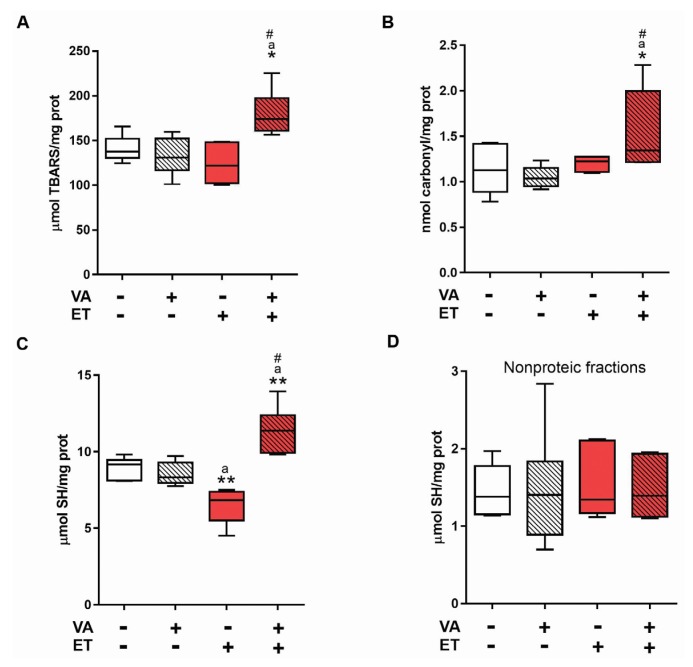
Effects of exercise and vitamin A supplementation on skeletal muscle oxidative damage markers. Data presented as box (median) and whiskers (interquartile interval) diagram (*n* = 6–8). (**A**) lipid peroxidation; (**B**) protein carbonylation; and (**C**,**D**) sulfhydryl group content. VA: vitamin A; ET: exercise training. * *p* < 0.05; ** *p* < 0.01 significant difference from sedentary group; ^a^ *p* < 0.01 significant difference from sedentary + vitamin A group; ^#^ *p* < 0.05 significant difference from exercise training group using one-way ANOVA followed by Tukey’s post hoc test.

We investigated the effect of vitamin A on skeletal muscle, as this is the tissue with the highest oxidative and stress-related demands during exercise training [46]. Muscle lipoperoxidation levels (Figure 3A) and protein carbonylation (Figure 3B) were increased in the ET + VA group, with a significant difference compared to the other three groups. These results indicate that VA supplementation causes oxidative damage to lipids and proteins in skeletal muscle of animals subjected to ET. Regarding sulfhydryl group content, the ET group exhibited a significant decrease in total thiol content (Figure 3C), which was significantly reversed in the ET + VA group. This result indicates that animals receiving VA supplementation and subjected to ET display an increased content of proteins with reduced thiol groups. Non-protein sulfhydryl levels did not show a significant difference between groups (Figure 3D).

#### 3.3.2. Antioxidant Enzyme Activity

**Figure 4:**
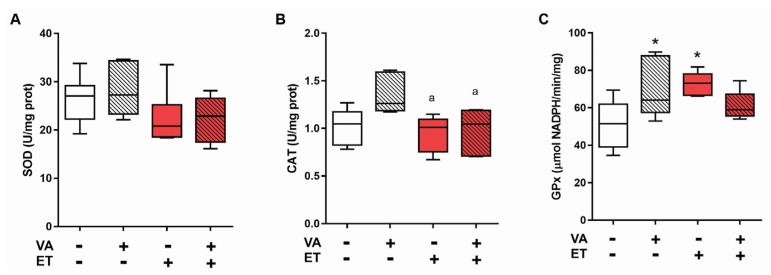
Effects of exercise and vitamin A supplementation on skeletal muscle antioxidant enzyme activity. Data presented as box (median) and whiskers (interquartile interval) diagram (*n* = 6–8). (**A**) Superoxide dismutase; (**B**) Catalase; and (**C**) Glutathione Peroxidase. VA: vitamin A; ET: exercise training. * *p* < 0.05 significant difference from sedentary group; ^a^ *p* < 0.05 significant difference from sedentary + vitamin A group using one-way ANOVA followed by Tukey’s post hoc test.

SOD activity did not present differences between groups (Figure 4A). CAT activity only differed in the group SE + VA, with a significant increase compared to ET and ET + VA (Figure 4B). On the other hand, GPx activity was significantly higher in SE + VA and ET groups compared to SE (Figure 4C).

#### 3.3.3. Antioxidant Enzyme Content Evaluated Using Western Blotting

**Figure 5:**
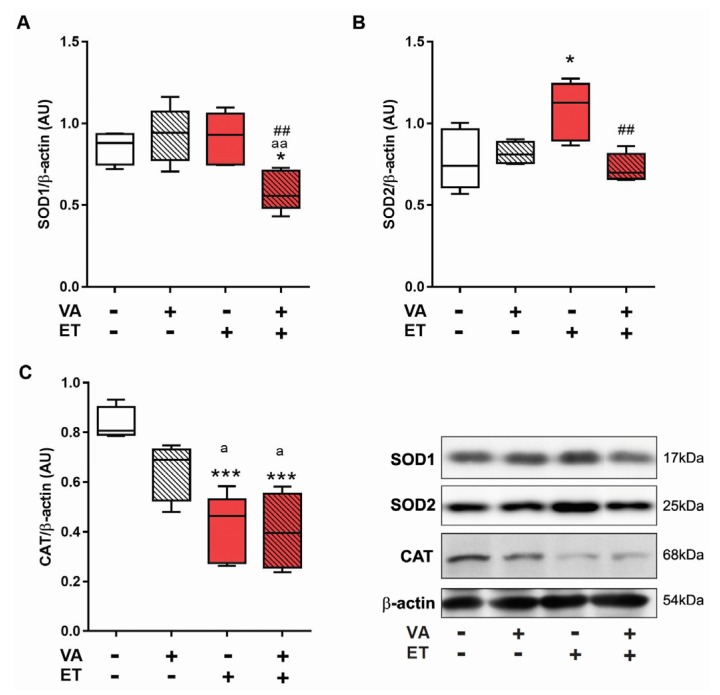
Effects of exercise and vitamin A supplementation on skeletal muscle antioxidant content. Data presented as box (median) and whiskers (interquartile interval) diagram (*n* = 6). (**A**) Superoxide dismutase-1; (**B**) Superoxide dismutase-2; and (**C**) Catalase content. VA: vitamin A; ET: exercise training. * *p* < 0.05; *** *p* < 0.001 significant difference from sedentary group; ^a^ *p* < 0.05; ^aa^ *p* < 0.01 significant difference from sedentary + vitamin A group; ^##^ *p* < 0.01 significant difference from exercise training group using one-way ANOVA followed by Tukey’s post hoc test. Representative Western blots are shown.

With no alteration in SOD and CAT activities, the next step was to determine through Western blotting the content of enzymes CuZnSOD (SOD1), the isoform present in cell cytoplasm, MnSOD (SOD2), the isoform located within the mitochondria [47], and CAT. Exercise training by itself did not increase the content of SOD1 in skeletal muscle (Figure 5A). However, the ET + VA group displayed significantly lower levels of SOD1 compared to SE and ET groups. SOD2 content (Figure 5B) increased significantly in the ET group compared to SE and ET + VA groups, though supplementation reversed this increase, as SOD2 content in the ET + VA group was significantly lower than in the ET group. CAT content in skeletal muscle (Figure 5C) decreased significantly in both exercise training groups compared to SE and SE + VA groups.

#### 3.3.4. Inflammation Marker Content Evaluated Using Western Blotting

**Figure 6:**
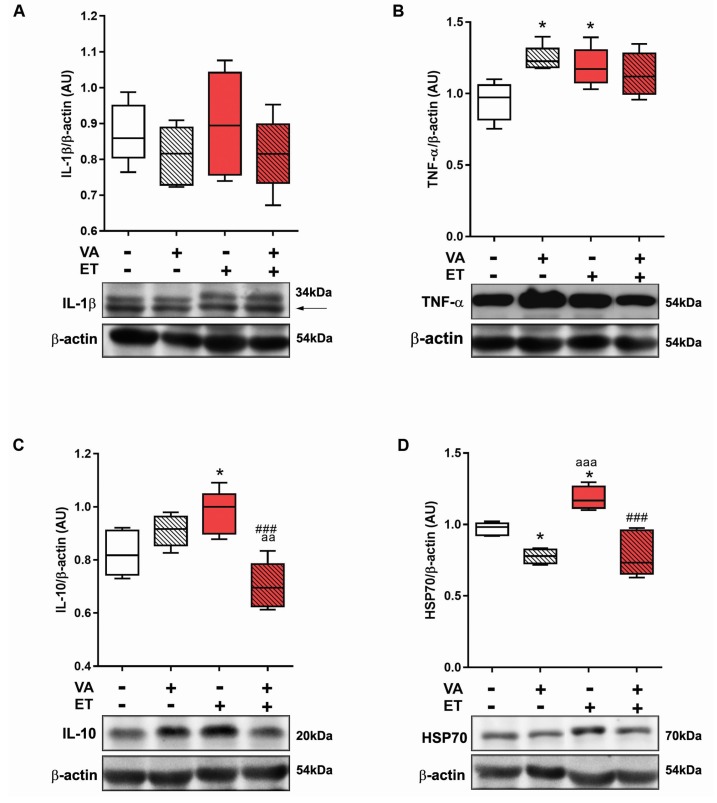
Effects of exercise and vitamin A supplementation on skeletal muscle inflammation marker content. Data presented as box (median) and whiskers (interquartile interval) diagram (*n* = 6). (**A**) Interleukin-1β; (**B**) Tumor necrosis factor-α; (**C**) Interleukin-10; and (**D**) Heat shock protein 70 content. * *p* < 0.05 significant difference from SE group; ^aa^ *p* < 0.01; ^aaa^ *p* < 0.001 significant difference from SE + VA group; ^###^ *p* < 0.001 significant difference from ET group using one-way ANOVA followed by Tukey’s post hoc test. Representative Western blots are shown.

Taking into consideration skeletal muscle oxidative damage results, we next evaluated the levels of inflammatory and stress markers IL-1β, TNF-α, IL-10, and HSP70. IL-1β values did not differ between groups (Figure 6A). TNF-α content increased in SE + VA and ET groups compared to SE (Figure 6B). IL-10 increased significantly in the ET group compared to SE and ET + VA groups (Figure 6C). HSP70 content was significantly lower in both vitamin A groups compared to the ET group, which had increased HSP70 content compared to SE (Figure 6D).

## 4. Discussion

Exercised animals exhibited a plateau in body weight gain, without influence from VA supplementation. This result is corroborated in the literature, as swimming exercise training with overload stabilizes weight gain in rats [35]. To assess tissue damage in serum, we measured the enzymatic activity of CK and LDH. When exercise intensity surpasses the capacity of muscle cell metabolism, membrane permeability increases and enzymes present in the cytosol leak into the extracellular environment [48]. In our study, the levels of LDH increased in both exercised groups, with no difference between them (Table 2). Regarding CK release, no differences between groups were detected. One explanation for this result is the timing of sample collection; the literature reports that, even with intense exercise, high levels of CK may not be detected when sample collection occurs after 24 h following the last training session [49]. TRAP serum results revealed that the ET group had higher antioxidant capacity compared to both sedentary groups, increasing more than twofold (Figure 1A), which agrees with the literature [50]. On the other hand, although the ET + VA group also showed a difference from SE, its antioxidant capacity was significantly lower compared to the ET group, indicating that VA impaired the total antioxidant capacity improvement resultant from the exercise itself. Cooperation among antioxidants in the blood circulation is conducted through redox reactions—for example, the ability of erythrocytes to regenerate ascorbic acid to ascorbate through ferricyanide reduction [51]. As the chemical structure of vitamin A is known to have an effect on redox reactions [24], it may have acted in a non-beneficial direction, reducing the total antioxidant capacity acquired with exercise training. 

The exact profile of cytokines released in response to exercise depends on the particular aspects of training, such as type, intensity, and duration [13]; nutritional issues [14,52]; and blood flow [53]. Results from this study showed increased levels of IL-1β in both exercise groups (ET and ET + VA) and increased TNF-α in all groups when compared to SE (Figure 2A,B). IL-1β and TNF-α are substantially present after long endurance bouts of exercise [53], as was applied in this study. The increase in TNF-α (Figure 2B) and the decrease in anti-inflammatory IL-10 (Figure 2C) observed in the SE + VA group indicates that vitamin A alone affected the immune response. Cytokines work synergistically to regulate the inflammatory cascade, and these results suggest that vitamin A by itself held up the basal inflammatory response in sedentary animals. Modulation of levels of circulatory cytokines by exercise or diet supplementation may take place due to changes in the inflammatory state of a variety of tissues, including adipose and liver tissues [3]. Here, we observed that muscle cytokine levels varied in response to exercise and VA supplementation, suggesting that the modulation of circulatory cytokines is influenced by cytokine production in muscle. This is further discussed below. 

Serum findings indicated that vitamin A did not have any protective or beneficial effects during or following exercise; taking this into consideration, we decided to evaluate the skeletal muscle, the tissue most under demand and affected by exercise training. For oxidative stress analysis, TBARS, carbonylation, and sulfhydryl residues were analyzed (Figure 3) [9]. The ET + VA group displayed significant increased lipid and protein damage, which did not happen in the ET group. Previous studies investigating the effects of supplementation on exercise showed no effect of vitamins in preventing oxidative damage [16,17]. Vitamin A, by its structure and potential free radical quenching action, apparently induced more oxidative stress in the skeletal muscle, leading to tissue damage. Regarding proteins that were oxidatively modified, the ET group exhibited a significant decrease in total thiol content, likely indicating elevated levels of glutathione disulfide (GSSG). GSSG is often employed as a sign of system’s response to oxidative stress, as its detection indicates that GSH groups are being actively involved in redox reactions [54]. Moreover, when tissue goes through intense oxidative stress, as provided by high-intensity exercise training, depletion of GSH within the cell is commonly observed [55]. Indeed, GPx activity increased in the ET group (Figure 4C). The ET + VA group showed higher levels of total thiol content, although TRAP assay results indicate that this group had lower serum antioxidant capacity. However, TRAP evaluates total antioxidant capacity, which, in the serum, is not exclusively comprised of thiols, but also phenols, ascorbic acid, and uric acid, among others [37]. Activity of the antioxidant enzymes SOD and CAT was also measured in skeletal muscle (Figure 4A,B), and activity did not show any difference between SE and exercised groups. In another study, a moderate swimming exercise protocol also displayed no difference in SOD activity in the skeletal muscle [56]. High-intensity exercise, like the activity performed in our study, induced no difference in aorta CAT activity, although SOD activity was greater in the exercised group [50]. Furthermore, in this study, tissue collection was performed 24 h after the last bout of exercise training. Some studies collect samples up to 2 h after the last bout, when the antioxidant system is working at its maximum and differences in enzymatic activity can be easily detected [57]. 

In order to clarify whether upregulation of endogenous antioxidant enzymes did occur, we performed Western blotting for SOD1 (Cu-ZnSOD), the isoform localized on cell cytosol; SOD2 (MnSOD), the isoform localized inside cell mitochondria [58,59]; and CAT within skeletal muscle (Figure 5). The expression of SOD1 did not change with exercise only; however, the ET + VA group presented a lower content of SOD1, which may be behind the elevated levels of oxidative tissue damage seen in this group. SOD2 content increased in the ET group, with a significant difference compared to SE and ET + VA groups. Studies with animals exposed to chronic exercise training showed an upregulation of mitochondrial SOD and GPx when compared to sedentary animals, thus presenting lower oxidative stress [55]. In this study, SOD2 content was higher only in the ET group. This may be one explanation for the lack of enhanced SOD activity, as specifically in this tissue, SOD2 only contributes 15%–35% of all SOD cell content [60]. CAT expression was lower in both exercise groups compared to the SE group, justifying the lower enzyme activity results. Literature findings regarding skeletal muscle CAT are controversial, as there is no consensus on the true effect of chronic exercise training. Some studies reported increased [61], decreased [6,62], or unchanged [63] CAT activity in response to exercise. Furthermore, ET and ET + VA groups were not different regarding CAT activity or expression, indicating no effect of vitamin A on the response of CAT to chronic exercise training. Studies addressing the effects of vitamin A supplementation and exercise training are rare, although VA is frequently associated with oxidative stress prevention. A recent study from 2016 evaluated the effects of four weeks of VA supplementation and changes in circulatory redox parameters in healthy young male subjects [64]. Using a daily dose of 30,000 RE (considered very high), parameters including lipid peroxidation, NO production, GSH levels, and antioxidant enzyme activity showed no difference from non-supplemented subjects. However, this study was conducted with sedentary individuals, and the training protocol was performed once a week over four weeks, using a protocol of exhaustion, different from a daily 60 min moderate to high-intensity exercise such as swimming training. Our study shows that VA supplementation causes oxidative stress in trained animals, and human studies using the same models will clarify the reproducibility of these results. Besides this study, no other works on the effects of VA on exercise training have been performed so far. 

Regular physical activity combined with a healthy diet is known to maintain a tissue anti-inflammatory phenotype [3]. Cytokines IL-1β, IL-10, and TNF-α are expressed in skeletal muscle and are increased upon exercise training; furthermore, both interleukins respond to a TNF-α stimulus [65]. While IL-10 acts as a highly effective anti-inflammatory agent, inhibiting the expression of pro-inflammation mediators [3], IL-1β induces pro-inflammatory events, and is related to pain susceptibility [66]. TNF-α was increased in the skeletal muscles of rats in the SE + VA and ET groups; however, this did not affect IL-1β protein content in any group (Figure 6). Interestingly, IL-10 levels increased only in the ET group, with a statistical difference from SE + VA and ET + VA. Exercise training may raise the levels of TNF-α, but adaptive responses, such as a greater expression of anti-inflammatory cytokines, also occur. This was not true in the ET + VA group, indicating that VA impaired this beneficial aspect of exercise training. In exercise training studies, it is usual to measure circulatory markers for stress, but the circulatory profile may be influenced by tissues other than muscles, which could complicate the interpretation of the results. One example is adipose tissue, which is very important when it comes to exercise training [67]. Obesity leads to an increase in circulatory pro-inflammatory cytokines by stimulating a pro-inflammatory state in adipose tissue, and healthy habits tend to prevent this by inhibiting the inflammation in this tissue [3]. The circulatory cytokine levels may indicate that inflammation is occurring in some tissues, and analyzing cytokine levels in specific tissues helps to clarify their origin.

Finally, Western blotting for HSP70 revealed differences between all groups, with upregulation of protein expression in the ET group and downregulation in vitamin A groups SE + VA and ET + VA. HSP70 is one component of a stress protein family that has increased expression as a cellular defense strategy. The literature describes that basal HSP70 expression occurs in athletes as well as healthy subjects, with no difference in quantity between type I and type II muscle fibers [68]. During exercise training, the cell environment undergoes changes in homeostasis, such as redox imbalance, high temperature, hypoxia, and glucose depletion. This kind of stress enhances tissue HSP70 levels, providing a cytoprotective effect [69] that includes prevention of oxidative damage and repair of proteins damaged by muscle contractions [70]. Our study revealed that vitamin A supplementation induced tissue oxidative damage and downregulation of endogenous antioxidant defenses in skeletal muscle of trained rats, which is likely to be related to suppression of HSP70 expression. Furthermore, VA combined with chronic exercise training inhibited the increase of IL-10 in skeletal muscle levels, blunting the anti-inflammatory cytokine response caused by exercise. Other studies have described that antioxidant supplementation impairs HSP70 synthesis induced by exercise [71], which leads us to believe that redox-dependent mechanisms are responsible for HSP70 downregulation, since VA is also considered an antioxidant molecule. The VA dose utilized in this study was based on the daily recommendation for human adult daily ingestion and considered the fact that the standard laboratory food provided to the animals contained a mix of vitamins including VA at a dose to fulfill the daily requirement for this vitamin. Higher or lower doses may show different effects than the ones presented here. Higher doses would probably provoke more tissue damage and inflammation as observed previously [26,27,28], and lower doses may show no effect at all, as many of the effects observed here could be considered mild. Exercise training itself is already a major source of ROS production, hence higher doses of VA were not considered for this study, as a synergistic pro-oxidant effect could take place, since excessive doses cause oxidative stress. The combination of VA intake from supplementation and food applied here is very likely to exceed the daily recommended amount, as often occurs with regular intake of diet supplements, but does not characterize hypervitaminosis, since it is below the tolerable upper intake level of 3000 RE/day for VA and acute toxic effects were not observed.

## 5. Conclusions

In conclusion, our results show that, despite its antioxidant status, vitamin A supplementation induces the release of stress markers, redox imbalance in serum, tissue damage, impaired antioxidant capacity, and inflammation in the skeletal muscle, probably due to inhibition of HSP70 expression in trained Wistar rats.

## Figures and Tables

**Table 1:** Effects of chronic exercise training and vitamin A supplementation on total body weight.

	SE	SE + VA	ET	ET + VA
Initial Weight (g)	337.6 ± 21.9	345.8 ± 24.3	350.5 ± 25	336.1 ± 27.2
Final Weight (g)	440.4 ± 25.2	456.9 ± 25.3	403.3 ± 26.9	398 ± 31.1
Δ weight gain (g)	99.3 ± 10	101.1 ± 16	68.7 ± 11.8 **^,aa^	66.6 ± 16.4 **^,aaa^

Data presented in mean ± standard error (SEM) (*n* = 6–8). SE: sedentary; SE + VA: sedentary + vitamin A; ET: exercise training; ET + VA: exercise training + vitamin A. ** *p* < 0.01 and significant difference from SE group; ^aa^ *p* < 0.01; ^aaa^ *p* < 0.001 significant difference from SE + VA group using one-way ANOVA followed by Tukey’s post hoc test.

**Table 2:** Effects of chronic exercise training and vitamin A supplementation on serum tissue damage markers.

	SE	SE + VA	ET	ET + VA
LDH	43.7 ± 1.18	49.5 ± 1.78	57.1 ± 0.08 *	68.7 ± 2.4 **^,a^
CK	315.8 ± 3.6	273.1 ± 2.8 *	304.8 ± 4.4	290.9 ± 5

Data presented in mean ± SEM (*n* = 6–8). LDH: lactate dehydrogenase; CK: creatine kinase. LDH and CK values expressed as U/L. SE: sedentary; SE+VA: sedentary + vitamin A; ET: exercise training; ET + VA: exercise training + vitamin A. * *p* < 0.05; ** *p* < 0.01 significant difference from SE group; ^a^ *p* < 0.05 significant difference from SE + VA group using one-way ANOVA followed by Tukey’s post hoc test.
